# Conducting Cancer Research among Culturally and Linguistically Diverse Groups in Australia: A Reflection on Challenges and Strategies

**DOI:** 10.3934/publichealth.2016.3.460

**Published:** 2016-07-08

**Authors:** Cannas Kwok

**Affiliations:** School of Nursing and Midwifery, Western Sydney University, Locked bag 1797, Penrith, 2751, New South Wales, Australia

**Keywords:** recruitment, minority groups, cancer research, challenges

## Abstract

**Background:**

While effort has been made to include minority groups in cancer studies in Western countries, overseas experience indicates that recruiting immigrant members to participate in cancer research is challenging. The aim of the paper is to set out the challenges and strategies needed to ensure the success of cancer research among culturally and linguistically diverse (CALD) groups in Australia.

**Discussion:**

Author suggested that partnership with ethnic community organization, research team, research setting and data collection methods, access to national data in cancer register, the informed consent process, and resources management are important elements to ensure the success of cancer research among CALD groups.

**Summary:**

The paper provides health care professionals with insight into methodological and practical issues needed to plan and conduct cancer research among CALD populations not only in Australia but also other Western countries, where numbers of minority groups are increasing significantly.

## 1. Introduction

Over the last few decades, immigration from less economically developed countries to Western countries has increased markedly. Currently, around 50% of all immigrants worldwide reside in ten highly urbanized, high-income countries such as Australia, Canada and the United States [1]. In Australia, more than a quarter (27.7%) of the population is overseas-born, 18% of these being from non-English speaking backgrounds [2]. One consequence of this demographic transition has been epidemiological transition of cancer prevalence. International studies reveal that Asian women in the USA have a 60% greater risk of breast cancer than their counterparts in Asian countries [3] while Chinese women in Australia have a 40% greater risk [4]. The added concern for immigrant women was being consistently reported having low participation rates in cancer screening in their adopted countries [5]–[7]. Studies have further demonstrated that cancer patients from minority cultures often experience a poor quality of life in the survivorship stage [8]. All these issues challenge health care professionals to gain population-specific knowledge of cancer prevention, screening, treatment and survivorship care. While efforts have been made to include minority groups in cancer studies, overseas experience indicates that recruiting immigrant members to participate in cancer research is difficult [9]–[11].

Drawing on the author's experiences gained from conducting numerous cancer research among culturally and linguistically diverse (CALD) groups in Australia, it is believed that perceptions of and willingness to participate in cancer research among such CALD groups are strongly influenced by cultural and social contexts. This paper aims to share the challenges and also what has been learned about strategies needed to ensure the success of the overall research project among these groups.

## 2. Partnership with Ethnic Community Organizations

Consistent with international studies conducted among immigrant groups [11]–[13] we found that establishing partnerships with ethnic community organizations (ECOs) was critical to the success of our research projects. The creation of such partnerships was made possible by the fact that in Australia, as in other countries, immigrants often seek to ‘find their feet’ by joining ECOs. Thus these organisations constitute one of the best platforms for recruiting people from CALD backgrounds in research. We proved that using the means described in this paper, we were able to recruit an optimum number of participants (approximately 2000) from the Chinese, Indian, African, Korean and Arabic communities in Australia to explore their cancer screening behaviours and cancer experiences.

Moreover, in line with the study by Wallington et al [11], we established that engagement with ECOs can promote the sustainability of research outcomes. Thus, in collaboration with a number of these organisations, we have been able to implement several cancer prevention and screening promotion programs on an ongoing basis. What is noteworthy in this regard is the way in which leaders of ECOs have continued our initial efforts to empower their community members through continuous training and education and thus sustain long-term behavioural change.

We further discovered that working closely and collaboratively with ECOs requires the application of a core set of strategies and that key elements needed to build up partnerships include *engagement, ongoing support, resourcing and acknowledgment of their roles* in the research.

*Community engagement* is a planned process with the specific purpose of working with identified groups of people, who are connected by special interests to address issues affecting their well-being [14]. The first step in this process is to establish relationships with ‘gatekeepers’, in this case the leaders of ECOs, to gain access to the target population. Since great store is set on interpersonal relationships in minority cultures [15], initial contact with the ECO leaders was usually made through face-to-face meetings between them and the researchers in our team who spoke their language and came from similar cultures. During these encounters, the aims of the research, details of how it would be conducted and clear explanations of recruitment criteria were provided. This direct, interpersonal contact served to establish trusting relationships.

Our experience supports the assertion of Fenlon and colleagues [16] that engaging the gatekeepers as early as possible in the planning stage is very important. It was observed that the gatekeepers serve two main functions which can facilitate research efforts. Firstly, their role as community leaders enhanced the credibility of the research team and secondly they provided invaluable advice about the cultural context of the community and the needs of its members. For example, their warning that Chinese people do not like to discuss health issues around Chinese New Year resulted in recruitment for cancer research being avoided around that time.

Consistent with the suggestion made by Casado and colleagues [14], we also determined that establishing a common goal between gatekeepers and researchers in the planning stage is highly beneficial. Sharing with community leaders the ultimate goal of our research, namely cancer prevention and the promotion of quality of life and psychosocial wellbeing after cancer diagnosis, generally created a climate of co-operation. We observed that the more understanding the leaders had of the goals of the research project, the more they engaged with ‘hearts and minds’ as allies in the recruitment process. We received a remarkable amount of positive feedback from the leaders who obviously appreciated the opportunity to collaborate and this was also true of the memberships of the various ECOs.

*Ongoing support* — As most ECOs are run by volunteers who have limited resources, the researcher should aim to minimize any extra workload the project could entail. To maintain engagement, it is vital for the researcher to provide ongoing support during the recruitment process. This could take form of regular email and telephone contact throughout the set-up and recruitment period, providing updates and promptly addressing any queries about the recruitment process.

Providing *adequate resources* to support the recruitment process should be included in the research plan. The researchers should bear in mind that ECOs, which usually have very limited financial resources, will not be able to bear or even share the costs of recruitment. Thus the research team should budget to pay for items like phone calls, stationery, photocopying and the costs of sending information to participants through the post.

To maintain close and long term relationships with ECOs, public *acknowledgement* of their assistance in the project is vital. Such acknowledgement should include a thank-you letter and also appear in the final report or paper resulting from the research, a copy of which should be sent to the ECO.

## 3. Research Team

Research among CALD groups calls for researchers to possess or develop special qualities. Obviously it is highly beneficial when researchers are equipped with background knowledge of the cultural norms and practices of the target group. We agree with Karwalajtys and colleagues [12] that having a bilingual and bicultural researcher in the team is indispensable for conducting cancer research among CALD groups. In the study we conducted among Chinese-Australian women for example, all qualitative interviews were conducted by the author who shared the language and cultural heritage of her informants. Since the topic of cancer was known to be sensitive in Asian and Arabic cultures [17],[18], it was vital to conduct the interviews in a culturally appropriate manner. Thus the researcher was careful to avoid using taboo terms such as ‘death’ as it indicates bad luck in Chinese culture [19]. Similarly, when we recruited women from Arabic, Indian, Korean and African cultures to participate in the surveys, the invitation often started with the words: “Would you like to fill in a breast health survey?” instead of ‘breast cancer survey’.

Being able to conduct interviews in the preferred language of the informants is another major advantage for the researcher as had been shown overseas studies conducted among immigrants [9],[12]. Thus, even though some of our informants were fluent in English, all interviews were conducted in their first language since it was clear to us that ease and familiarity with the language in which interviews were conducted, enhanced the willingness of informants openly to share their narratives and opinions. Moreover researchers using the vernacular are likely to have a better understanding of the slang and cultural idioms that the participants very often use. In cancer research, it is an advantage to know that an ordinary conversation can contain many terms which need to be understood in a cultural context. For example, because many Chinese regard the word ‘cancer’ as a bad omen, they avoid using it, preferring euphemisms such as ‘lump’ or ‘tumour’ or the typical Chinese term *nham*, meaning ‘growth’. In fact Cantonese-speaking doctors commonly use this lay term when discussing cancer with patients whereas a researcher without any Chinese background would very likely not be able to understand or respond to the use of this term.

## 4. Research Setting and Data Collection Methods

Interviewing people about sensitive topics requires special skills and techniques [20]. Studies have revealed that Chinese women associate breast cancer with immoral behaviour and that cancer is regarded as a stigmatizing disease [17]. Such ideas can reduce their willingness to participate in cancer research. Taking this sensitivity into account, we found that it is particularly important to build a rapport by using tactful, euphemistic terms when talking to subjects prior to the interview. This is in line with Liamputtong's finding [21] that creating a close relationship with an interviewee can encourage them to impart in-depth information in qualitative interviews.

In our study exploring breast cancer experiences among Chinese women, the process of building rapport and relationships of trust can be said to have started in the recruitment phase in which initial contacts were made by the ECO leaders. As has been demonstrated in overseas studies many minority groups respond favourably to direct contact from known individuals [13]. We also found that starting off the in-depth interview by sharing personal stories or experiences about having a family member or relative diagnosed with cancer or about being the carer of a cancer patient, was an effective way of earning an interviewee's trust. We found that a researcher who was also a health professional or more particularly a nurse with extensive experience of caring for cancer patients and their families, helped informants to feel that their needs and feelings were being understood and that they were thus more forthcoming with information useful to the study.

In keeping with the findings of Halcomb et al [22] which indicated that focus groups are an effective way of engaging people from CALD groups, we chose to use this means for data collection rather than individual interviews for exploring breast cancer experiences among Chinese women. Even though this was outside the Chinese cultural norm in terms of which people seldom talk about issue like cancer outside the family [15] we found that because participants in our study had similar experiences, the focus groups indeed created a non-threatening environment in which participants felt free to discuss the sensitive issue of breast cancer. The appropriateness of the venue and the setting for focus groups can also enhance the engagement of informants [21]. To ensure the research setting was informal, relaxing, and welcoming, our focus groups met in a Chinese cultural centre which the informants were familiar with and in which they felt comfortable.

**Figure 1.**
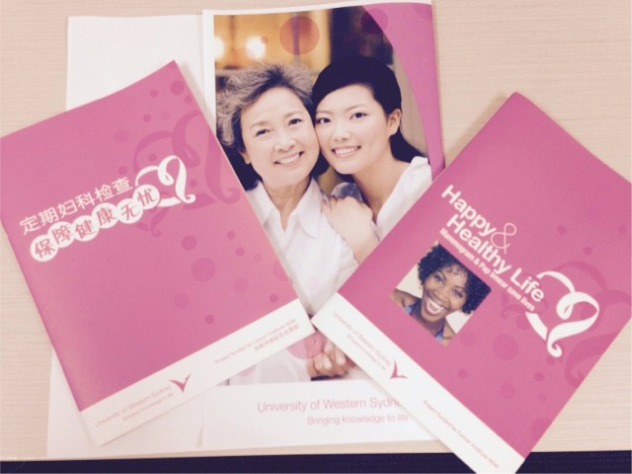
Culturally sensitive cancer screening promotional resources developed for Chinese and African communities.

The literature documents that language barriers are one of the key barriers to the recruitment of minorities in cancer studies [9],[11]. Thus, providing research materials in the informants' native language is essential. However, we argue that direct translation of existing promotional materials is unlikely to fit into the belief system of the CALD groups because the content, for instance pictures of Caucasian women, does not acknowledge minority cultural norms and their beliefs about cancer. One of those beliefs is that only Caucasian women get breast cancer and to counteract that idea, the research materials we developed used contained relevant images of women designed to ensure that immigrant women could associate them with breast cancer (See figure 1).

## 5. Access to National Data in the Cancer Register

Representative samples play a major role in credibility of research findings to inform evidence-based practices. Accessing the target sample for a population-based study, particularly CALD groups, is extremely challenging in Australia. In our quantitative study of which investigated breast cancer experiences among Chinese-Australian women, 1,574 patients were identified in the New South Wales cancer registry database. In order to reach them, the first step was to obtain permission from their oncologist or family doctor so as to ensure the patient was mentally and physically fit to participate in the study. Despite invitation letters and two reminders sent to oncologists and family doctors, only 15% (222) replied. Among their patients, only 78 Chinese-Australian women agreed to participate in the study, and the resultant 5% response rate provided a sample too small for analysis. Our experience was consistent with overseas studies, in which physicians were found to be the major barrier in the recruitment process for cancer studies [9],[16] because some of them did not see the significance or value of the research for their patients. We recommend efforts should be made to improve doctors' knowledge of and attitudes towards cancer research and the significant role they can play in recruiting subjects for study.

## 6. The Informed Consent Process

Studies by Karwalajtys et al [12] indicate that the complexities of consent forms and procedures prevented some minority populations from participating in research. Even though all the materials used in our studies were translated into the participants' native language, we found that obtaining a signed informed consent form was challenging. It became evident that some Chinese and Korean participants had no understanding of the need and purpose of the consent procedure because they perceived signing a consent form as ‘commitment’ or ‘obligation’. This indicates that although the informed consent process should not be compromised, some flexibility in requiring written consent may be needed. One thing observed during our studies was that verbal consent using an audio-tape was more acceptable and comprehensible to the participants than written consent. This form of obtaining consent was therefore used, even though it was more time consuming. Our experience indicates that it is essential to emphasise to possible participants that the intention of the informed consent process is to protect their rights and does not place them under any obligations. Finally, it is important to note that having a bilingual researcher who can communicate with participants in their native language greatly facilitates the consent process.

## 7. Resources Management

Previous studies among immigrant groups have reported unexpected increase in timelines and budgets [12],[16] and in this regard we can confirm that bilingual research requires significant additional funding and is very time consuming. Even with the assistance of an ECO for recruitment, the data collection for our study of the breast cancer screening beliefs among women from among Indian-, Korean-, African-, Arabic- and Chinese-Australian women had to be extended from nine to 15 months. This made it important to pay special attention to maintaining the spirits and the energies of the research team and community partners.

Extra funding was also essential. One unanticipated financial demand was imposed by necessity for obtaining the word-by-word transcriptions essential in qualitative research [21]. Data in languages other than English needs to be translated but this had its advantages because the translation process enabled other team members to participate and assist in the minimisation of any interpretation bias. To ensure lexical equivalence and reconciliation, a back translation technique was required [23], in terms of which the English version was translated back into the original language to ensure the original meanings had been adequately reflected by someone who was probably providing a professional translation service. This process is extremely time consuming and costly. On average, preparing just one hour's worth of interview data (transcribed, translated and back-translated) for data analysis can take up to 25 hours and cost US $800.

## 8. Implications for Practice

**Figure 2.**
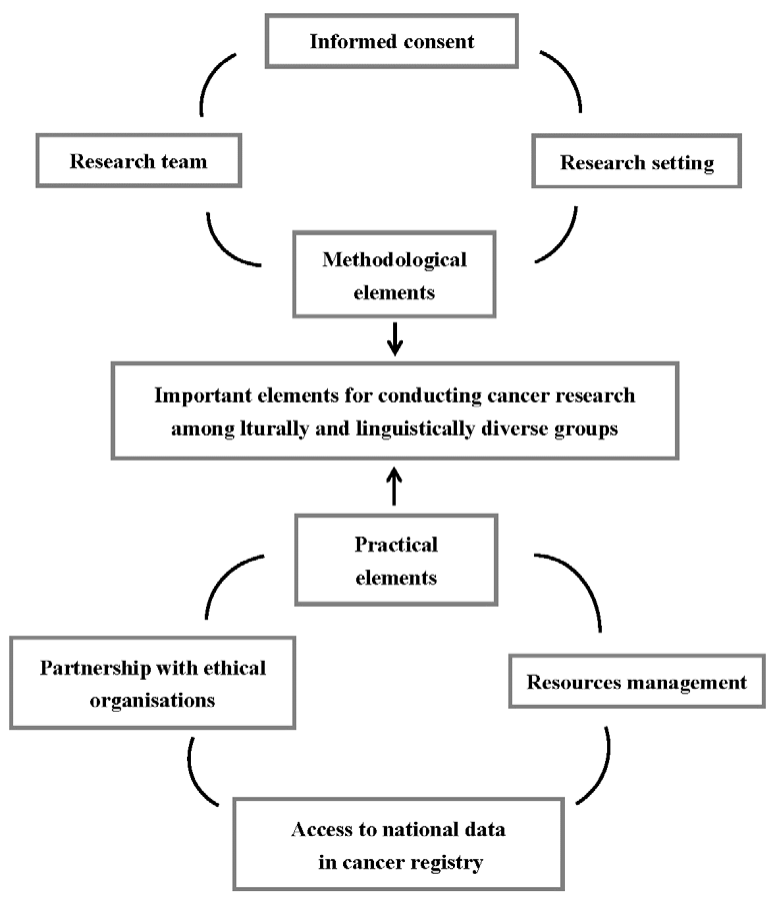
The important elements to ensure the success of conducting cancer research among culturally and linguistically diverse groups.

To increase the participation of minority groups in cancer research, it is essential for researchers to acquire the cultural competence to enable them to develop strategies for identifying and understanding the unique issues faced by target populations. Moreover it should be remembered that when developing a particular cultural competency, the result is not likely to be a case of ‘one size fits all’; we are conscious of the fact that the strategies we used among Chinese, Indian, Korean, African and Arabic communities may not be suitable for use among other CALD groups. Nevertheless, this paper, we believe, can form the basis for providing health care professionals with insight into methodological and practical issues needed to plan and conduct cancer research among CALD populations not only in Australia but also other Western countries where the numbers of minority groups are increasing significantly. The important elements to ensure the success of conducting cancer research among culturally and linguistically diverse groups are summarised in figure 2.
